# 33rd Brazilian Society for Virology (SBV) 2022 Annual Meeting

**DOI:** 10.3390/v15040943

**Published:** 2023-04-10

**Authors:** Maite Freitas Silva Vaslin, Gustavo Peixoto Duarte da Silva, Alessandra Alevato Leal, Larissa Mayumi Bueno, Cíntia Bittar, Gabriela Fabiano de Souza, Karine Lourenço, Maria Isabel Maldonado Coelho Guedes, José Luiz Proença-Módena, João Pessoa Araújo Júnior, Helena Lage Ferreira, Flávio Guimarães da Fonseca

**Affiliations:** 1 Departamento de Virologia, Instituto de Microbiologia Paulo de Góes, Universidade Federal do Rio de Janeiro (UFRJ), Rio de Janeiro 21941-599, Brazil; gustpdsilva@gmail.com; 2 Laboratório de Pesquisa em Virologia Animal, Departamento de Medicina Veterinária Preventiva, Escola de Veterinária, Universidade Federal de Minas Gerais (UFMG), Belo Horizonte 31270-901, Brazil; alessandraalevato@hotmail.com (A.A.L.); mariaisabel.guedes@gmail.com (M.I.M.C.G.); 3 Departamento de Medicina Veterinária, FZEA-USP, Universidade de São Paulo, Pirassununga 13635-900, Brazil; larissambueno@gmail.com (L.M.B.); hlage@usp.br (H.L.F.); 4 Departamento de Biologia, Instituto de Biociências Letras e Ciências, Universidade Estadual Paulista (UNESP), São José do Rio Preto 15054-000, Brazil; cintia.bittar@unesp.br; 5 Departamento de Genética, Evolução, Microbiologia e Imunologia, Instituto de Biologia, Universidade Estadual de Campinas (UNICAMP), Campinas 13083-862, Brazil; gabriela.sfabiano@gmail.com (G.F.d.S.); jlmodena@gmail.com (J.L.P.-M.); 6 Instituto de Biotecnologia (IBTEC), Universidade Estadual Paulista (UNESP), Botucatu 18607-440, Brazil; karine_lourenco@hotmail.com (K.L.); joao.pessoa@unesp.br (J.P.A.J.); 7 Instituto de Ciências Biológicas (ICB), Universidade Federal de Minas Gerais (UFMG), Belo Horizonte 31270-901, Brazil

**Keywords:** Brazilian Society for Virology, SBV, SBV annual meeting, human virology, veterinary virology, plant virology, invertebrate virology, basic virology, environmental virology

## Abstract

Each year, the Brazilian Society for Virology promotes a national meeting during the second semester of the year. In October 2022, the 33rd meeting took place at Arraial da Ajuda, Porto Seguro, Bahia, in-person:.this was the first in-person meeting since 2019, as the 2020 and 2021 events occurred online due to the issues imposed by COVID-19. It was a great pleasure for the whole audience to return to an in-person event, which certainly improved the interactions between the attendees in all ways. As usual, the meeting involved massive participation of undergraduate, graduate, and postdoc students, and several noteworthy international researchers were present. During five afternoons and evenings, attendees could discuss and learn about the most recent data presented by distinguished scientists from Brazil and other countries. In addition, young virology researchers from all levels could present their latest results as oral presentations and posters. The meeting covered all virology areas, with conferences and roundtables about human, veterinary, fundamental, environmental, invertebrate, and plant virology. The costs associated with attending the in-person event caused a slight reduction in the number of attendees compared to the two online events. However, even with this issue, the attendance was impressive. The meeting successfully achieved its most important goals: inspiring young and senior scientists and discussing high-quality, up-to-date virology research.

## 1. Introduction

For the last 33 years, the Brazilian Society of Virology (SBV) has been organizing annual national meetings, bringing together the best senior scientists in Brazil, renowned researchers in the field worldwide, and young virology researchers and students. Young researchers in the area and undergraduate and graduate students are encouraged to actively participate in these meetings, making these events an important forum for discussion and inspiration for all young Brazilian virologists to learn about the most recent data and results in the area.

As a national society, SBV meetings cover five main areas of interest: human, veterinary, plant and invertebrate, environmental, and basic virology, and the events are held in different regions of Brazil each year.

In 2022, it was a great pleasure to hold an in-person event. After two years of online events, the contact between colleagues and collaborators was refreshing after days of tension and isolation. The 33rd SBV National Congress was held in Arraial da Ajuda, in Porto Seguro district, Bahia state. Almost all the speakers were present, but some lectures were remotely delivered. 

Four hundred eighteen attendees were at the 33rd SBV National Meeting, with a makeup of 57% students and 43% professionals, including 44 postdoctoral researchers. This year, the event started on 17 October and ended on 21 October. A new format was applied, with the SBV supporting a parallel event, the 9th Annual International Experimental Biology and Medicine Conference (IEBMC 2022). This new format was a great success and amplified the visibility of both events, providing an exciting forum for the attendees of both meetings. 

Compared to the last two SBV annual meetings, the 33rd SBV meeting had fewer attendees from all categories. This reduction can be attributed to the high costs of an in-person meeting, including airplane tickets, hotel reservations, and other expenses associated with accessing and staying at the event location. Brazil is a vast country, and the distances between the most important research centers and the event location could impose high travel costs. Nevertheless, even with this lower number of attendees, the 33rd SBV meeting could be considered a successful event in a difficult time for Brazilian science. 

## 2. 33rd SBV Annual Meeting Scientific Program

In 2022, the SBV meeting scientific program included eight plenary conferences, of which four were “state-of-the-art” talks, two technical conferences, and one special talk. In addition, the event had nine roundtables, nine oral presentation sessions shared between human, plant, veterinary, invertebrate, environmental, and basic virology, the Helio Gelli Pereira award session, and a precongress workshop with a total of 86 speakers. Among the speakers, six were from the USA, one from the Czech Republic, two were from Germany, one was from Norway, one was from the United Kingdom, and the remaining speakers were from Brazil. 

More than 21 researchers from all virology areas, mainly from Brazil, collaborate with the event, sharing roundtables and conferences, discussing the scientific program, evaluating poster submissions and/or presentations, and selecting the best submissions to receive the Helio Gelli Pereira award. Once again, SBV acknowledges the important work of these enthusiastic virologists. 

A total of 332 poster abstracts were submitted for the meeting, of which 331 were presented during the event as complete reports. This year, the event had posters presented by students and professionals from Paraguay (10), Colombia (1), Chile (1), Canada (1), and the Czech Republic (1), in addition to those from Brazil. 

Detailed information about the SBV 33rd meeting can be found at https://sbv.org.br/event/ (accessed on 20 February 2023) [1] and at https://sbv.org.br/files/anais_2022.pdf (accessed on 20 February 2023) [2].

### 2.1. Meeting Attendants

**Figure 1:**
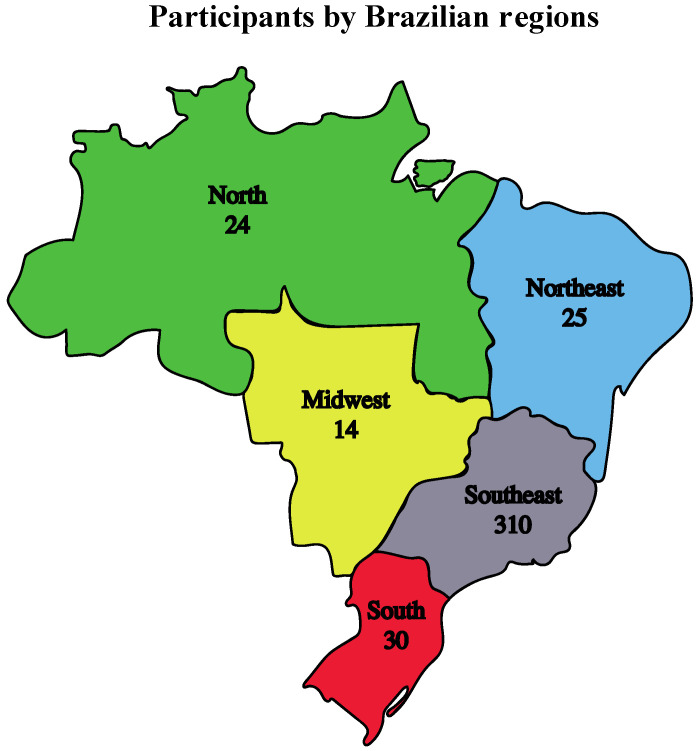
Distribution of the 418 Brazilian attendees at the 33rd SBV Annual Meeting by Brazilian geographic areas. Each Brazilian region is represented by a different color, and the number of attendees per region is shown. Nineteen attendees were foreign and were not represented on the map.

In 2022, the SBV annual meeting had a total of 418 participants, including professionals, undergraduates, and graduate students from all Brazilian regions (Figure 1) and other countries. 

**Figure 2:**
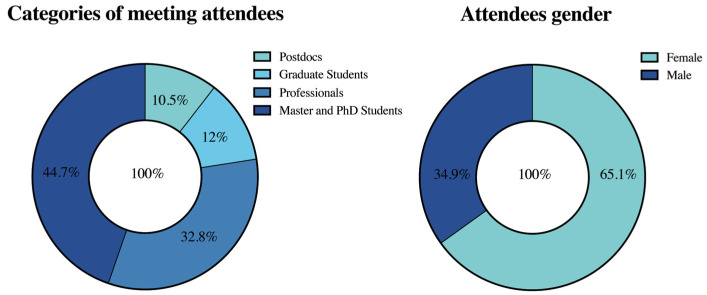
Percentage of attendees by categories (**left**) and by gender (**right**).

From this total, professionals represented 32.8% and students from all categories represented 67.2% of the meeting attendees (Figure 2). Of the students, 12% were undergraduates, 44.7% graduate students, and 10.5% were postdocs. As highlighted before, high attendance of students was achieved, showing that this annual meeting is succeeding in impacting young virologists from distinct parts of Brazil. Most attendees were women, representing 65.1% of all 33rd SBV meeting members. SBV is glad to see the increasing contribution of women in life sciences in Brazil. However, professional opportunities do not seem to follow this phenomenon since most of the lectures were given by men (59% men vs. 41% women). There are many competent and brilliant female researchers in virology, but this is not reflected in the meeting presentations of our virology society. This bias must be eliminated in the future.

### 2.2. Scientific Program

During the five days of the 33rd SBV meeting, the activities started at 1 p.m. and finished at 9:30 p.m. (Table 1). During the morning, the conference rooms held the IEBMC 2022, which started at 8 a.m. and finished around midday. This kind of schedule permitted the attendees of the 33rd SBV to attend all the conferences and roundtables during the morning events. 

### 2.3. Conference Speakers and Roundtable Presentations

The opening conference, titled “Friend and foe: the complex interactions between dengue and Zika virus immune responses and epidemiology,” was presented by Dr. Eva Harris from the University of California, Berkeley, CA, USA. In her very enthusiastic talk, Dr. Harris showed a comprehensive overview of years of information collected from health centers in Nicaragua, showing how infections, reinfections, and cross-infections from both viruses can impact patient intake. 

Before the opening ceremony, a precongress course on “Science communication and divulgation” was conducted by Dr. Laura M.A. de Oliveira and Prof. Tatiana de C.A. Pinto, both from the Universidade Federal do Rio de Janeiro, UFRJ, Brazil. 

On the second day of the meeting, Dr. Colleen B. Jonsson from the University of Tennessee Health Science Center, Memphis, TN, USA, presented a conference that brought together interests from both of the simultaneous congresses. The conference’s title was “Disease, ecology, and evolution of hantavirus in South America.” On the same afternoon, a state-of-the-art conference on fundamental or basic virology was presented by Dr. Akira Ono from the University of Michigan, Ann Arbor, MI, USA. Dr. Ono’s research focused on virus-cell interactions in HIV infections, and his conference title was “The roles played by the plasma membrane components in HIV-1 assembly and beyond.” Finally, after three simultaneous oral presentations, it was time for the first round table, called Young Inspiring Researchers. This roundtable has become a tradition in the SBV meeting since it first started some years ago and has been a great success. Young Brazilian researchers showing emerging skills are invited to present their latest research data, an event that works to stimulate the careers of young virologists. This year, Dr. Flávio L. Matassoli, from NAID/NIH, Maryland, USA, and Dr. Luciana P. Tavares, from Harvard Medical School, Boston, MA, USA, presented the talks “SARS-CoV-2 protein vaccination elicits long-lived plasma cells in *Rhesus* macaques” and “Pro-resolving therapies for Influenza A virus disease”, respectively. Both talks were fascinating and excited the audience.

Closing the second day, a special talk, titled “Multidisciplinary research with arboviruses at the Brazilian synchrotron source,” was presented by Dr. Rafael Elias, from CNPEM, Campinas, SP, Brazil. The conference was exciting and brought the audience up-to-date with data from a multidisciplinary approach to help understand arboviruses. 

The third meeting day started with a state-of-the-art conference on environmental virology. Dr. Rodrigo F. de Bueno from UFABC, Santo André, SP, Brazil, talked about the contamination of wastewater with SARS-CoV-2 in Brazil, and his conference title was “Wastewater-based epidemiology for SARS-CoV-2: Lessons learned from recent studies by the wastewater COVID-19 monitoring network—MCTI.”

Next, there were two simultaneous roundtables. One roundtable’s focus was basic virology. In this roundtable, Dr. Luciana B. de Arruda, from UFRJ, RJ, Brazil, exposed the latest results discovered by her team in Zika virus IFN response activation. Her conference title was “Activation of microvascular endothelial cells and type INF response in resistance and tolerance against Zika infection.” In the same round table, Dr. Eugênio Hottz, from UFJF, Juiz de Fora, MG, Brazil, presented the talk “Thromboinflammation in COVID-19 mechanisms and contributions to pathogenesis and Dr. Enrique M.B. Pierulivo, from ICB, USP, São Paulo, SP, Brazil, gave the talk “Cell transformation by human papillomaviruses: from the nucleus to the extracellular matrix and back.”

The parallel roundtable focused on plant virology. In this roundtable, Dr. Juliana de Freitas Astúa, from EMBRAPA Mandioca e Fruticultura, Cruz das Almas, BA, Brazil, presented the talk “Updates on the citrus leprosis virus C-plant interaction.” Dr. Alice Inoue-Nagata, from EMBRAPA Hortaliças, DF, Brazil, gave the talk “Critical points for a virus control strategy via application of dsRNA molecules,” and Dr. Elizabeth P.B. Fontes, from UFV, Viçosa, MG, Brazil, gave the talk “Begomoviruses NSP-host interactome: integrating developmental signals, antiviral immunity, and pro-viral functions.”

Two technical conferences were presented during the event. The first one, titled “lllumina genomic surveillance of infectious diseases,” was presented by Dr. Michelle G. Penna from Illumina Co. and occurred after the conference given by Dr. Colen Jonsson. The second technical conference, “xGenTM amplicon panels for metagenomics and viruses: investigative answers to your questions,” was presented by Síntese Biotecnologia Co. and occurred after the two roundtables described above. 

The fourth day of the event began with a state-of-the-art conference on veterinary virology, where Dr. Edviges M. Pituco, from PAHO/PANAFTOSA OIE Reference Laboratories, Brazil, presented the talk “Updates and advances in the control of foot-and-mouth disease in Brazil.” The conference followed with one roundtable on human virology (“Epidemiology and evolution of viruses in the context of One Health”) and another on environmental virology. Three exciting talks took place at the human virology roundtable: “SARS-CoV-2 and other respiratory viruses: from surveillance to pandemic action in the context of One Health,” presented by Dr. Edison L. Durigon, ICB USP, São Paulo, SP, Brazil; “Characterization of Ilheus virus: implications for emergence,” presented by Dr. Nikolaos Vasilakis, UTMB, Texas, USA; and “Emergence, spread, and evolution of SARS-CoV-2 lineages circulating in Brazil during the first 18 months of the pandemic,” presented by Dr. Gonzalo B. Bentacor, Fiocruz, Rio de Janeiro, RJ, Brazil. The environmental virology roundtable started with the talk by Dr. Caroline Rogotto, Univ. Feevale, Novo Hamburgo, RS, Brazil, titled “Environmental surveillance as a complementary tool for monitoring COVID-19,” and was followed by the talk, “The influence of *Escherichia coli* phage vB_EcoM-UFV13 on a consortium of sulfate-reducing bacteria opens a new window to bacteriophage use” presented by Dr. Roberto S. Dias, UFV, Viçosa, MG, Brazil. Moreover, the last talk was presented by Dr. Gabriel M.F. Almeida, UiT at the Artic University of Norway, Norway, with the title “The forgotten tale of Brazilian phage therapy.” This last talk was presented as a video conference. 

After the roundtables, another state-of-the-art conference was presented to the whole meeting audience, named “Plant manipulation by geminiviruses” by Dr. Rosa Lozano-Dúran from the Department of Plant Biochemistry, Centre for Plant Molecular Biology (ZMBP), Eberhard Karls University, Tübingen, Germany. 

The last day of the meeting began with three simultaneous roundtables. One dedicated to veterinary virology involved Dr. Jan Drexler Felix, Universitätsmedizin Berlin, Berlin, Germany, presenting “Challenges toward serologic diagnostics of emerging arboviruses.” The following talks were “Monkey see, monkey do: potential zoonotic viruses in nonhuman primates from Southern Brazil,” presented by Dr. Fernando R. Spilki, Feevale, Novo Hamburgo, RS, Brazil, and “Point-of-care diagnostic platforms for arboviruses,” presented by Lindomar J. Penna, Fiocruz-PE, Recife, PE, Brazil. In parallel, an invertebrate virology roundtable occurred. In this roundtable, the talks “*Spodoptera frugiperda* fall armyworm virus and its biological control applications” by Dr. Leonardo A. da Silva, AgbiTech, Goiania, GO, Brazil, “Regulation of dengue transmission by the natural mosquito virome” by João Marques Trindade, UFMG, Belo Horizonte, MG, Brazil, and “One bacterium in the fight against arboviruses” by Luciano Moreira, Fiocruz, Belo Horizonte, MG, Brazil, were presented. The third roundtable of the day focused on basic virology and included the following talks: “Antagonism of nuclear antiviral responses by herpesviruses,” presented by Dr. Colin Crump, University of Cambridge, United Kingdom; “Immune responses to the efferocytosis of SARS-CoV-2-infected dying cells,” presented by Dr. Larissa D. Cunha, USP, SP, Brazil; and “Unique structural features of flaviviruses’ capsid proteins and their role in viral capsid assembly,” presented by Dr. Andrea da Poian, UFRJ, Rio de Janeiro, RJ.

Dr. Felipe Naveca (Fiocruz, Manaus, AM) (human virology), Dr. Sergio de Paula (Universidade Federal de Viçosa—UFV, Viçosa, MG) (environmental virology), Dr. Juliane Deise Fleck (Universidade Feevale, Nova Hamburgo, RS) (environmental virology), Dr. Iranaia Assunção Miranda (Universidade Federal do Rio de Janeiro, Rio de Janeiro, RJ) (basic virology), Luis Lamberti Pinto da Silva (FMRP, Ribeirão Preto, SP) (basic virology)*,* Dr. Paula Rahal (IBILCE-Universidade Estadual Paulista—UNESP, São José do Rio Preto, SP) (human virology), Dr. Eurico Arruda (Faculdade de Medicina de Ribeirão Preto, USP, SP) (human virology), Dr. Tatiana Domitrovic (UFRJ, RJ) and Dr. Daniel Ardisson-Araujo (Universidade de Brasilia, Brasília, DF) (plants and invertebrate virology), Dr. Abelardo Silva Jr (UFV, Viçosa, MG) (veterinary virology), Marcelo de Lima (UFPel, Pelotas, RS) (veterinary virology), and Matheus Weber (Feevale, Nova Hamburgo, RS) (veterinary virology) were the chairs of the conferences and roundtables. 

### 2.4. Abstracts, Oral Presentations, and the Helio Gelli Pereira Award

**Figure 3:**
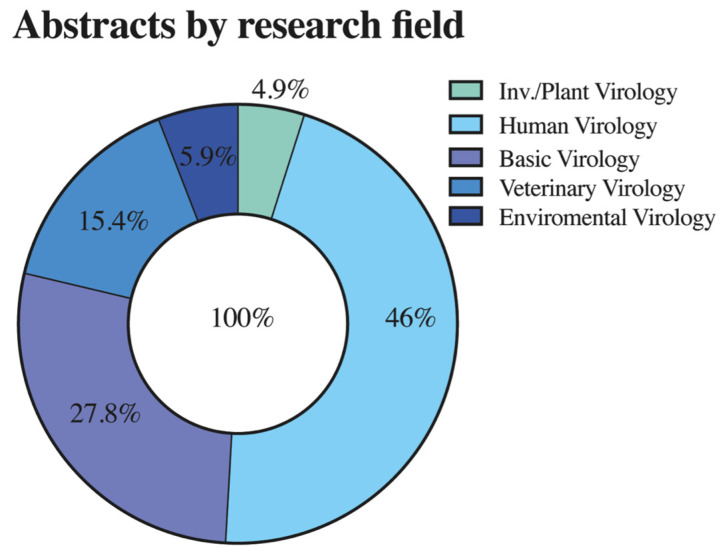
Abstracts presented at the 33rd SBV Annual Meeting, distributed by their interest area.

A total of 331 abstracts were approved to be presented at the meeting. As usual, human virology represented the majority with 149 abstracts, representing 46.0% of the total number of posters, followed by basic virology (90 abstracts, 27.8%), veterinary (50 abstracts, 15.4%), environmental (19 abstracts, 5.9%), and plant and invertebrate virology (16 abstracts, 4.9%) (Figure 3).

Among the 331 abstracts, 45 studies were selected by 12 senior researchers to be presented as short oral presentations. Nine oral presentation sections were presented in the 33rd SBV meeting: three for human virology, with fifteen students in total; two for basic virology (with five students); one for veterinary virology (with five students); two for plant and invertebrate virology (with ten students); and one for environmental virology (with five students) (Table 2). The studies selected for oral presentation were performed in 18 independent institutions: 17 from three distinct Brazilian regions and one from Paraguay (Universidad National de Assumption). Among Brazilian institutions, the highest number of selected studies came from the Universidade Federal do Rio de Janeiro (UFRJ), followed by the Universidade Federal de Minas Gerais (UFMG).

Each oral presentation section was evaluated by at least two independent researchers who evaluated each talk and selected the best presentation in each section. The winners of the best presentation of each section are highlighted in Table 2 with an asterisk.

All of the remaining abstracts were presented as posters that were evaluated individually by a special scientific commission.

Respecting the tradition of SBV meetings, in 2022 the Helio Gelli Pereira (HGP) award was given to the best complete scientific articles produced by virology students. To participate, candidates had to apply and submit their work to a scientific committee composed of distinguished virologists from different areas. This year the commission was composed of Nikolaos Vasilaskis, from the University of Texas Medical Branch at Galveston, USA; Jan Drexler Felix, from Universitätsmedizin, Berlin, Germany; Juliane Deise Fleck, from Univ. Feevale, Novo Hamburgo, RS; Luis Lamberti da Silva, FMRP/USP, Ribeirão Preto, SP; Daniel M.P. Ardisson-Araujo, UnB, Brasília, DF; and José Luís Proença Módena, Unicamp, Campinas, SP.

Seven articles were selected to be presented orally during the SBV meeting. During this session, named the HGP award, the committee chose the best articles or presentations in each category (given by undergraduate and graduate students) (Table 3). 

The HGP award for the undergraduate student category was given to Luan Rocha Lima, from UFRJ, RJ, Brazil, for the work “Differential modulation of type I IFN response by distinct Zika virus isolates impacts virus replication and disease tolerance in vitro and in vivo.” The graduate student award was conferred upon Otávio Augusto Chaves, from Fiocruz, RJ, who presented the work “Commercially available flavonols are better SARS-CoV-2 inhibitors than isoflavone and flavones.” *Viruses*, published by MDPI, the American Society for Microbiology (ASM), and SBV support the HGP award. In 2022, ASM granted one eBook and a one-year membership for the winners, and *Viruses* offered a full waiver for publication by both research groups awarded. SBV partnerships with *Viruses* represent a significant incentive for students and their research groups, especially in Brazil, where very few institutions can support APC fees.

## 3. Conclusions

The 33rd SBV meeting marks the return of SBV in-person meetings. It was a great pleasure for all the attendees to finally return to the presential form, with the participation of influential scientists in the virology area, undergraduate and graduate students, and a large group of Brazilian virology researchers together at the event. The meeting occurred in a very beautiful and pleasant region of Brazil, south of Bahia state, more precisely at Arraial da Ajuda, Porto Seguro city, which has gorgeous beaches with warm waters. In this unique scenario, the meeting talks and sessions included massive participation from attendees every day, who enjoyed the high-quality virology science from all virology areas that were discussed. 

The cost of attending an in-person meeting was prohibitive for some of the potential attendees and caused a reduction in the number of attendees compared to the 2020 and 2021 online events. Despite this slight reduction in attendee numbers, the meeting was a great success. One of the positive points of the 2022 meeting was pairing with the 9th IEBMC. The sharing of the venue permitted some of the attendees of the SBV meeting to also attend IEBMC sessions during the mornings. To encourage students to take part in both events, the subscription for one event permitted participation in both.

## Figures and Tables

**Table 1:** Scientific programming schedule of the 33rd Brazilian Society for Virology Congress.

Activities	Subject
17 October—Monday	
Workshop	Pre-congress workshop: Divulgamicro
Opening Ceremony	SBV President welcome to the participants
Opening Conference	Friends and foe: the complex interactions between Dengue and Zika virus immune responses and epidemiology
Opening Cocktail	Opening cocktail to welcome all attendees
18 October—Tuesday	
Conference	Joint SBV and IEBMC Conference: Disease, ecology, and evolution of hantaviruses in South America
Technical Conference	Illumina genomic surveillance of infectious diseases: strategies to identify novel strains and other emerging pathogens
State-of-the-art Basic Virology	The roles played by the plasma membrane components in HIV-1 assembly and beyond
Oral Presentation 1	Human Virology 1
Oral Presentation 2	Plant and Invertebrate Virology
Oral Presentation 3	Environmental Virology
Roundtable 1	Young Inspiring Researchers
Special Talk	Multidisciplinary research with arboviruses at the Brazilian synchrotron source
Poster Session 1	Even numbers
19 October—Wednesday	
State-of-the-art Environmental Virology	Wastewater-based epidemiology for SARS-CoV-2: Lessons learned from recent studies by the wastewater COVID-19 monitoring network—MCTI
Roundtable 2	Basic Virology
Roundtable 3	Plant Virology
Technical conference 2	xGen^TM^ amplicon panels for metagenomics and viruses: investigating answers to your questions
Oral Presentation 4	Human Virology 2
Oral Presentation 5	Basic Virology
Oral Presentation 6	Plant and Invertebrate Virology
Roundtable 4	Human Virology
Poster Session 2	Odds numbers
20 October—Thursday	
State-of-the-art Veterinary Virology	Updates and advances in the control of foot-and-mouth disease in Brazil
Roundtable 5	Human Virology
Roundtable 6	Environmental Virology
State-of-the-art Plant and Invertebrate Virology	Plant manipulation by geminiviruses
Oral Presentation 7	Human Virology 3
Oral Presentation 8	Basic Virology 2
Oral Presentation 9	Veterinary Virology
Hélio Gelli Pereira Award	Oral presentations
Closing Party	Closing party
21 October—Friday	
Roundtable 7	Veterinary Virology
Roundtable 8	Invertebrates Virology
Roundtable 9	Basic Virology
Awards announcement	Announcement of the best poster and oral presentations and the Hélio Gelli Pereira Award
SBV General Assembly	Closing session

**Table 2:** Oral presentations at the 33rd Brazilian Society for Virology Congress.

First Author and Institution	Title
Human Virology 1	
Kíssila Rabelo (UERJ, RJ) *	SARS-COV-2 is persistent in the placenta and causes macroscopic, histopathological, and ultrastructural changes [3]
Giovana Waner Carneiro de Almeida (UFABC, SP)	Expression patterns of HCMV IL-10 transcripts in cells with different permissivity for HCMV and in GBM tissues [4]
Tárcio Peixoto Roca (Instituto Oswaldo Cruz, RJ)	The SARS-CoV-2 Omicron Variant of Concern and its rapid spread throughout the Western Brazilian Amazon [5]
Sarah Aparecida Rodrigues Sérgio (Centro de Tecnologia em Vacinas/UFMG, MG)	Comparison of pseudovirus neutralization assays with PRNT and indirect ELISA in a follow-up study of a longitudinal cohort of COVID-19 patients [6]
Ellen Viana de Souza (Instituto Adolfo Lutz, PA)	Human bocavirus epidemiology from historical fecal samples in Brazil, 1998–2008 [7]
Plant and Invertebrate Virology 1	
Igor da Silva Teixeira (Faculdade De Medicina De São José Do Rio Preto, SP)	Detection of insect-specific viruses in aedes and culex mosquitoes collected in urban and forest fragment areas of northwest São Paulo state [8]
Luis Janssen Maia (Universidade De Brasília, DF) *	RNA Virome of sylvatic mosquitoes sampled in Cerrado regions of Minas Gerais [9]
Victória Bernardi Ciconi (Faculdade De Medicina De São José Do Rio Preto, SP)	First detection of the insect-specific viruses Humaiatá-tubiacanga virus in haemagogus mosquitoes collected in the Brazilian Amazon rainforest [10]
João Pedro Nunes Santos (Uesc, Ba)	Identification of endogenous viral elements in bee genomes [11]
Sabrina Ferreira de Santana (Uesc, Ba)	Investigating the virome of the *Theobroma cacao* pollinator *Aphis aurantii* [12]
Environmental Virology	
Ana Paula Correia Crispim (UFMG, MG)	The discovery of a new mimivirus isolate in association with virophage-transpoviron elements in Brazil highlights the main genomic and evolutionary features of this tripartite system [13]
Meriane Demoliner (Universidade Feevale, RS) *	Diversity of unknown gokushoviruses in human feces and surface water in southern Brazil [14]
Anderson Carvalho Vieira (UESC, BA)	Metavirome analysis of Carpotroche brasiliensis (Raddi) A. Gray (Achariaceae) in agroecological interface reveals viral diversity in cacao-cabruca agroforestry systems and in regions of the Brazilian Atlantic Forest [15]
Mônica Cristina de M. Silva (Instituto Evandro Chagas, MS)	Metagenomic analysis of the viral microbiota of Amazonian rivers located in the northeastern region of Pará state, Brazil [16]
Talita Bastos Machado (UFMG, MG)	Discovery and characterization of Cedratvirus pambiensis: a 1 micrometer giant virus infecting amoebas [17]
Basic Virology 1	
Gustavo Peixoto Duarte da Silva (UFRJ, RJ)	Autophagy regulation by early SARS-CoV-2 strains is pivotal for its maintenance [18]
Romulo Leão Silva Neris (UFRJ/RJ)	Immunoproteasome activation regulates viral replication and mediates muscle damage during arthritogenic alphavirus infection [19]
Juan Oswaldo Concha Casaverde (USP, SP) *	Rab27a is a host factor required for efficient OROV egress in mammalian cells [20]
Bruno Braz Bezerra (UFRJ, RJ) *	Hydroxpropyl-beta-cyclodextrin (HP-BCD) inhibits SARS-CoV-2 replication and virus-induced cytokines [21]
Rosa Maria Mendes Viana (USP, SP)	Study of permissiveness, expression of viral genes, and cellular effects of respiratory syncytial virus in CD4+ T lymphocytes [22]
Human Virology 2	
Ighor Leonardo Arantes Gomes (Fiocruz, RJ)	Comparative epidemic expansion of SARS-CoV-2 variants Delta and Omicron in the Amazonas, a Brazilian setting with high levels of hybrid immunity [23]
Ivana Preciosa Fernéndez Jara (Universidad Nacional de Asunción, Paraguay)	Variability of rotavirus VP7 and VP4 genes detected in stool samples of children under 5 years old with gastroenteritis in Asunción, Paraguay, during 2015–2019 [24]
Débora Familiar Rodrigues Macedo (Fiocruz)	Impact of the ChAdOx1 nCoV-19 vaccine on the modulation of inflammatory mediators involved in the clinical outcome of COVID-19 V [25]
Vitória Riquena Grosche (Unesp, SP)	Simultaneous determination of HCV genotype and NS5B resistance associated substitutions using dried serum spots from São Paulo state, Brazil [26]
Wallace Rafael Barbosa de Lima (UFRJ, RJ)	Zika virus infection disrupts the myogenic reparative program of skeletal muscle [27]
Plant and Invertebrate Virology 2	
Roy Bogardid Ardon Espinal (UESC, BA)	The complex virome associated with the cocoa pathogens *Ceratocystis cacaofunesta* and *Ceratocystis fimbriata* [28]
Lucas Antônio Stempkowski (UESC, BA)	Genetic variability and population structure of wheat stripe mosaic virus [29]
Alex Moura da Silva (UFRJ, RJ)	A case report of the emergence of cotton blue disease resistance-breaking viral isolates in a greenhouse in Brazil [30]
Anderson Carvalho Vieira (UESC, BA)	Metavirome analysis of *Carpotroche brasiliensis* (Raddi) *A. Gray* (*Achariaceae*) reveals a new virus from the genus Gammanucleorhabdovirus (Rhabdoviridae, -ssRNA) [31]
Ivair José de Morais Júnior (UNB, DF) *	Whole genome analyses suggest that potato and pepper PVY isolates are distinctly evolving [32]
Basic Virology 2	
Isabella Luiza Martins de Aquino (UFMG, MG)	Surface fibrils organization as specific trademarks of different mimivirus lineages [33]
Karine Lima Lourenço (UFMG, MG)	Zoonotic vaccinia virus strains belonging to different genetic clades exhibit immunomodulation abilities that are proportional to their virulence [34]
Leonardo Linhares Miler da Silva (UFRJ, RJ)	Chikungunya and Mayaro virus replication in skeletal muscle promotes chronic muscle atrophy and disruption in myogenesis triggered by inflammatory mediators [35]
Marina Alves Fontoura (UNICAMP, SP)	Usutu virus: another emergent flavivirus impacting pregnancy in a mouse model [36]
Álvaro Zocratto da Silveira e Silva	The effect of obesity and metformin on the response of murine macrophages to *in vitro* infection by Dengue virus [37]
Human Virology 3	
Andreza Parreiras Gonçalves (Fiocruz, MG) *	Evaluation of the systemic immune response of patients with yellow fever through a plaque reduction neutralization test (PRNT) using a wild yellow fever virus [38]
Renan Julio Mourão Ramos (UFRJ, RJ)	Activated macrophage controls CHIKV and MAYV skeletal muscle replication and preserves infected fiber structure in co-culture cell mode [39]
Michael Edward Miller (UNICAMP, SP)	Regulatory action of interferon-stimulated genes (ISGs) in oropouche virus infection [40]
Carolina Mattoso Lopes de Azevedo (IFRJ, RJ)	3D model of the placental barrier for functionality studies during ZIKV infection [41]
Adriana Luchs (Instituto Adolfo Lutz, SP)	Coxsackievirus A6 causing a hand-foot-and-mouth outbreak in Paraíba state, northeastern Brazil, 2018 [42]
Veterinary Virology	
Larissa Mayumi Bueno (USP, SP)	Alpha- and beta-coronavirus diversity in Brazilian bats [43]
Laura Morais Nascimento Silva *	The new subgenotype VI.2.1.2.1 of Newcastle disease virus in pigeons from Brazil is distinct from African viruses [44]
Forlan Fernandes de Jesus (IOC, Fiocruz)	Inclusion body disease in boa constrictor from Rio Grande do Sul, Brazil: a case report [45]
Erica Azevedo Costa (UFMG, MG)	Use of an inactivated recombinant vaccine against H1N1 swine influenza virus: immunization trial in a murine model [46]
Laís Santos Rizotto (USP, SP)	Analysis of the evolutionary profile of RDRP and NSP3 proteins of porcine epidemic diarrhea virus (PEDV) from samples obtained from *Tadarida brasiliensis* urban bats [47]

* Indicates the best presentations selected by scientific committee.

**Table 3:** Oral presentation during the HGP award session.

Hélio Gelli Pereira—Award Session	
Luan Rocha Lima (UFRJ, RJ) *	Differential modulation of type I IFN response by distinct Zika virus isolates impacts virus replication and disease tolerance in vitro and in vivo [48]
Roberta Salzone Medeiros (I. Adolfo Lutz)	Monitoring of rotavirus infection in domestic dogs and cats in Brazil during a 10-year follow-up (2012–2021): full genotype characterization of Brazilian canine G3P [3] strains [49]
Dayane Azevedo Padilha (UFSC, SC)	Emergence of two distinct SARS-CoV-2 gamma variants and the rapid spread of P.1-like-II SARS-COV-2 during the second wave of COVID-19 in Santa Catarina, Southern Brazil [50]
Juliano de Paula Souza (USP, SP)	Breastfeeding by Chikungunya virus-infected dams confers resistance to challenge in the offspring [51]
Keyla Santos Guedes de Sá (FMRP, SP)	Inflammasome activation in the pulmonary parenchyma defines two distinct profiles associated with cytokine storms and worsening of lung function in COVID-19 patients [52]
Mayara Mattos da Conceição (IOC)	Atazanavir is a competitive inhibitor of SARS-CoV-2 Mpro, impairing variants replication in vitro and in vivo [53]
Otávio Augusto Chaves (IOC Fiocruz, RJ) *	Commercially available flavonols are better SARS-CoV-2 inhibitors than isoflavone and flavones [54]

* Indicates the best presentations selected by HGR scientific committee.

## Data Availability

Not applicable.
